# Population genetics of a recent range expansion and subsequent loss of migration in monarch butterflies

**DOI:** 10.1111/mec.16592

**Published:** 2022-07-21

**Authors:** William B. Hemstrom, Micah G. Freedman, Myron P. Zalucki, Santiago R. Ramírez, Michael R. Miller

**Affiliations:** ^1^ Department of Animal Science University of California Davis California USA; ^2^ Department of Evolution and Ecology University of California Davis California USA; ^3^ Center for Population Biology University of California Davis California USA; ^4^ School of Biological Sciences The University of Queensland Brisbane Australia

**Keywords:** monarch butterfly, population genomics, range expansion, serial dispersal

## Abstract

Range expansions—whether permanent or transient—strongly influence the distribution of genetic variation in space. Monarch butterflies are best known for long‐distance seasonal migration within North America but are also established as nonmigratory populations around the world, including on Pacific Islands. Previous research has highlighted stepwise expansion across the Pacific, though questions remain about expansion timing and the population genetic consequences of migration loss. Here, we present reduced‐representation sequencing data for 275 monarchs from North America (*n* = 85), 12 Pacific Islands (*n* = 136) and three locations in Australia (*n* = 54), with the goal of understanding (i) how the monarch's Pacific expansion has shaped patterns of population genetic variation and (ii) how loss of migration has influenced spatial patterns of differentiation. We find support for previously described stepwise dispersal across the Pacific and document an additional expansion from Hawaii into the Mariana Islands. Nonmigratory monarchs within the Mariana Islands show strong patterns of differentiation, despite their proximity; by contrast, migratory North American samples form a single genetically panmictic population across the continent. Estimates of Pacific establishment timing are highly uncertain (~100–1,000,000 years ago) but overlap with historical records that indicate a recent expansion. Our data support (i) a recent expansion across the Pacific whose timing overlaps with available historical records of establishment and (ii) a strong role for seasonal migration in determining patterns of spatial genetic variation. Our results are noteworthy because they demonstrate how the evolution of partial migration can drive population differentiation over contemporary timescales.

## 1 INTRODUCTION

Over extended timescales, geographical range expansions generally involve decreasing relatedness and increasing contributions of genetic drift in populations further from the original source population (Excoffier et al., 2009; Hewitt, 1996). This pattern is especially evident in serial stepwise expansion events, in which populations are founded in a stepping‐stone fashion (Ibrahim et al., 1996; Slatkin & Excoffier, 2012). Serial dispersal is characteristic of many postglacial range expansions into temperate regions and has been shown for species including eider ducks (*Somateria mollissima*) (Tiedemann et al., 2004), ragwort (*Senecio helleri*) (Bettin et al., 2007), rough‐skinned newts (*Taricha granulosa*) (Kuchta & Tan, 2005) and European butterflies (Dapporto et al., 2019).

Studies on the population genetics of geographical range expansions tend to focus on expansion events that occur over extended timescales, though the same expansion processes characterize annual movements associated with seasonal migration. Unlike permanent range expansion, however, seasonal migration may involve individuals capable of making round‐trip journeys and traversing the entire species range in their lifetime (Dingle, 2014), thereby limiting opportunities for genetic divergence in allopatry. In species that migrate seasonally, patterns of population genetic variation in space are best captured by considering migratory connectivity of breeding populations (e.g., Cohen et al., 2018; Gao et al., 2020). For example, Wilson's warbler (*Wilsonia pusilla*) has eastern and western North American summer breeding populations that are genetically distinct, despite sharing an overwintering range in Central America (Irwin et al., 2011), and anadromous Coho (*Oncorhynchus tshawytscha*) and steelhead (*O. mykiss*) salmon populations show strong genetic differentiation corresponding to the river drainages where they spawn (Prince et al., 2017; Waples et al., 2004). By contrast, Japanese eels (*Anguilla japonica*) that share common spawning grounds but migrate to disparate areas across temperate Asia show little evidence for genetic differentiation over time or space (Gong et al., 2019).

Migratory species that show evidence for partial migration, whereby species comprise both migratory and nonmigratory populations (Chapman et al., 2011), often feature populations that are highly differentiated, both phenotypically (e.g., Altizer & Davis, 2010; Dingle et al., 1980) and genetically (e.g., Gómez‐Bahamón et al., 2020; Zhan et al., 2014). The phenomenon of partial migration is common across the tree of life and has been documented in birds (Adriaensen & Dhondt, 1990), insects (Menz et al., 2019) and ungulates (Berg et al., 2019). Although the evolutionary origins of partial migration are sometimes unclear, one recently invoked scenario involves a migratory, geographically widespread lineage giving rise to one or more nonmigratory descendant lineages that become genetically distinct due to mismatches in the timing and/or location of breeding. This scenario has been hypothesized to be an important contributor to patterns of speciation in tropical birds (e.g., Gómez‐Bahamón et al., 2020; Kondo et al., 2008) and may also contribute to diversification of other groups.

Monarch butterflies (*Danaus plexippus* [L.]) provide an intriguing system for studying the effects of both global range expansion and loss of migration on spatial population genetic structure. Migratory North American monarchs comprise a single genetically indistinguishable population (Lyons et al., 2012; Talla et al., 2020) and have a summer breeding range that covers most of the North American continent. Over recent evolutionary history, monarchs have expanded their range globally (Ackery & Vane‐Wright, 1984; Fernández‐Haeger et al., 2015; Pierce et al., 2015; Pierce, Zalucki, et al., 2014a; Vane‐Wright, 1993; Zalucki & Clarke, 2004; Zhan et al., 2014), with a southern expansion into South America and the Caribbean, an eastward expansion across the Atlantic and into the Iberian Peninsula, and a westward expansion across the Pacific. In contrast to their migratory North American ancestors, these expansion populations generally form nonmigratory, year‐round breeding populations in areas where they become established (Zhan et al., 2014). The exceptions to this pattern are in southern Australia and New Zealand, where monarchs move seasonally and form overwintering clusters akin to those observed in western North America (James, 1993; Wise, 1980). In this paper, we focus exclusively on the monarch's expansion and subsequent loss of migration in populations across the Pacific.

Little is currently known about how contemporary loss of migration has affected fine‐scale patterns of population differentiation in monarchs or other taxa (but see Samarasin et al., 2017). Two studies have indirectly addressed this question in monarchs: Hughes and Zalucki (1984) used four allozyme markers and found relatively high overall *F*_ST_ (0.032) between monarchs in host plant patches over small spatial scales (tens to hundreds of kilometres) in Queensland, Australia. Pierce, de Roode et al. (2014b) used microsatellite markers and showed that monarchs from the Hawaiian archipelago show little differentiation among islands. However, the conclusions of these studies are limited by the spatial scale of sampling and the number of loci studied. Furthermore, the timing of the monarch's Pacific expansion remains uncertain. Demographic simulations indicate that establishment timing may have happened as long as 2000–3000 years ago (Zhan et al., 2014), although these estimates conflict with historical records, which suggest that expansion across the Pacific happened between the years 1840 and 1900 (Freedman et al., 2020; Zalucki & Clarke, 2004). Further demographic modelling that accommodates a broad range of potential establishment scenarios—including variable establishment timing, founding population sizes and past changes in population size—might help to resolve the discrepancy between historical and model‐based estimates of expansion timing.

In this study, we sequenced 275 monarch butterflies at >70,000 highly variable genomic sites from the ancestral North American population and many Pacific Island populations, including a number of previously unsampled populations: the Mariana Islands (Guam, Rota and Saipan), Norfolk Island, Victoria and New South Wales. The goals of this study were to understand (i) overall patterns of genetic relatedness among Pacific and North American populations; (ii) the timing of range expansion and the amount of ongoing gene flow between North American and Pacific populations; and (iii) how migratory and nonmigratory populations differ in their distribution of population genetic variation in space.

## 2 METHODS

### 2.1 Sample preparation and sequencing

Monarchs were collected as either larvae or adult butterflies from various locations across their current geographical range between 1990 and 2017 (Table S1). DNA was extracted from samples using a magnetic bead‐based protocol (Ali et al., 2016) and quantified using Quant‐iT PicoGreen dsDNA Reagent (Thermo Fisher Scientific) on an FLx800 Fluorescence Reader (BioTek Instruments). Restriction‐associated digest (RAD) DNA libraries were then created using the *Pst*I restriction enzyme according to Ali et al. (2016) and sequenced using 150‐bp paired‐end sequencing on an Illumina Hi‐Seq 4000.

### 2.2 Sequence alignment, filtering and genotype calling

We aligned raw sequence data to version 3 of the monarch butterfly genome assembly (Zhan & Reppert, 2013) using the mem algorithm implemented in the Burrows–Wheeler Aligner (Li & Durbin, 2009). Sequence data were sorted and filtered for PCR (polymerase chain reaction) duplicates and improper pairs using samtools (Li et al., 2009). We first removed potential paralogous sites using the ngsparalog tool by removing all sites within 1 kb of any single nucleotide polymorphism (SNP) with a log ratio test statistic of >10 in any population (Linderoth, 2018). From this, we created five different data sets using different filtering schemes appropriate for different downstream analysis.

### 2.3 Data sets

1. For use in demographic reconstruction and directionality index (*ψ*) calculation, we called genotypes using the samtools genotype likelihood model (Li et al., 2009) as implemented in the angsd software package with a minimum mapping and base call quality score of 20, a SNP *p*‐value of 1e‐8, a uniform genotype prior and a posterior genotype probability cutoff of 0.95 (Korneliussen et al., 2014). To reduce potential bias due to linkage for demographic analyses, we randomly subsampled SNPs such that no locus was within 10,000 bp of another using a custom r script. The resulting SNPs were used to calculate site frequency spectra (SFS) and then projected down to a sample size of 100 gene copies from North America and 10 from Hawaii for demographic analysis and down to 10 gene copies in each population for calculation of *ψ* using the methods described by Gutenkunst et al. (2009). These projection numbers were picked to maximize the remaining number of SNPs in the data set. The SFS was polarized via reference to whole genome sequence data of the best‐sequenced individual of the monarch's sister species *Danaus erippus* (Zhan et al., 2014) by alignment to the monarch genome as described above. While we did not use a Hardy–Weinburg Equlibrium (HWE) filter here, only a very small proportion of our loci were consistently not in HWE across populations (*p* < 1 × 10^−6^ in only 86 out of 11,384 loci, calculated using the method of Wigginton et al., 2005).
2. For use in calculating basic diversity statistics (the average number of pairwise differences, or π, observed heterozygosity, or *H*_O_, and the ratio of within‐sample heterozygous to homozygous loci, or Het/Hom), the fixation index (*F*_ST_), and isolation‐by‐distance (IBD), we called genotypes as in data set 1, then removed individuals genotyped at <75% of loci. Since strong bottlenecks are likely to cause large differences in allele frequencies between populations, which, in conjunction with very different sample sizes between populations, can result in loci with very low overall minor allele frequencies having relatively high frequencies in individual populations, we did not use a minor allele frequency filter when calling genotypes. Some populations did not remain in the analysis after this filtering step.
3. To calculate Tajima's *D*, we implemented the same filtering steps as described for data set 2, but without using an SNP *p*‐value filter in angsd.
4. For analyses that did not require called genotypes (principal component analysis, or PCA, NGSadmix, and a neighbour‐joining tree), we used angsd as in data set 1, but did not call genotypes and instead estimated the likelihoods with a minor allele frequency filter of 0.05. For the PCA and neighbour‐joining tree, the input distance matrix was created using the Identity‐by‐State approach in angsd (Korneliussen et al., 2014). No individuals were removed for these analyses.

We also generated more thoroughly filtered versions of data sets 3 and 4 (see Supplementary Methods, Filtered Data sets), although these additional filtering steps did not meaningfully influence our inferences.

### 2.4 Patterns of relatedness among monarch populations

We calculated π, *H*_O_, Het/Hom and Tajima's *D* (Tajima, 1989) within each population using the snpr package (Hemstrom & Jones, 2021). We calculated *F*_ST_ between populations using the r implementation of the genepop software package (Rousset, 2008) with a minor allele frequency cutoff of 0.05 and bootstrapped individuals between populations randomly 1000 times to calculate *F*_ST_ significance levels using the snpr package (Hemstrom & Jones, 2021). For each of these statistics, the eastern and western North American samples were pooled together, based on results from previous studies (Lyons et al., 2012; Talla et al., 2020). To determine if our relatively light filtering approach biased our results, we also reran the diversity statistics π, *H*_O_, Het/Hom and Tajima's *D*, *F*_ST_, and IBD analyses using more heavily filtered data sets, as described in the Supporting Methods.

To describe the basic population structure, we created a neighbour‐joining tree (Saitou & Nei, 1987) using the ape r package version 5.0 (Paradis & Schliep, 2019) and conducted a PCA. For comparison, NGSadmix was used to generate individual ancestry coefficients for each individual for between one and nine putative population clusters (*k*) (Skotte et al., 2013). Each value of *k* was run 10 times, and the results were collapsed into consensus plots using clumpp (Jakobsson & Rosenberg, 2007). The pophelper (Francis, 2017) and snpr (Hemstrom & Jones, 2021) r packages were used to run these analyses. We used the method of Evanno et al. (2005) to detect the number of clusters present from the results; however, since this method has reproducibility issues (Gilbert et al., 2012), tends to underestimate the true *k* unless in the context of a complex hierarchical examination (Janes et al., 2017) and generally does not improve estimates of *k* (Waples & Gaggiotti, 2006), we also looked at patterns of clustering in general across a range of *k* values.

### 2.5 Serial expansion

To quantify the direction and strength of population spread across the Pacific, we calculated *ψ* (Peter & Slatkin, 2013) for each pairwise combination of North America, Hawaii, Queensland, Guam, Rota and Norfolk Island populations using the snpr package (Hemstrom & Jones, 2021).

### 2.6 Genetic variation across space in migratory vs. nonmigratory populations

We looked for evidence of IBD between samples from the Mariana Islands (nonmigratory), Hawaii (nonmigratory), Australia (partially migratory), and North America (migratory). To do so we calculated Edwards' angular genetic distance (Edwards, 1971) between each pair of samples from the given populations, and then compared these distances to the geographical distances between samples using a Mantel Test (Mantel, 1967). Here, we expect stronger patterns of IBD within locations where monarchs have ceased seasonal migration. As with *F*_ST_, we used data set 2 with an additional minor allele frequency cutoff of 0.05.

### 2.7 Demographic history of the monarch's expansion

To describe the patterns of establishment and migration between North America and the Pacific, the demographic reconstruction program δaδi (hereafter dadi, Gutenkunst et al., 2009) was used to estimate the demographic history of the North American and Hawaiian samples. Briefly, since demographic processes influence the frequency of common or rare alleles across loci, and the SFS describes how many individual loci fall into each possible allele rarity in each population, the SFS can be used to infer historical population processes. dadi therefore uses simulation to compare the SFS predicted under a specific demographic history to the SFS observed from the data in order to evaluate the likelihood of a demographic model and to optimize the parameters of that model. We chose to focus on Hawaii since previous work has suggested that this island group was probably the first in the Pacific colonized by monarchs (Zalucki & Clarke, 2004; Zhan et al., 2014), and thus the timing of the monarch introduction there represents the earliest possible time for any introductions in the Pacific. The general approach that we describe here is similar to that described in Zhan et al. (2014), but with a few key differences: (i) Zhan et al. (2014) pooled samples from six distinct Pacific populations for their analysis, while we only focused on the Hawaiian population; (ii) the parameter values reported in Zhan et al. (2014) represent a single instance of one focal demographic scenario, whereas our analyses considered a large set of candidate demographic scenarios and report results from multiple iterations of each scenario.

### 2.8 dadi model selection

**FIGURE 1:**
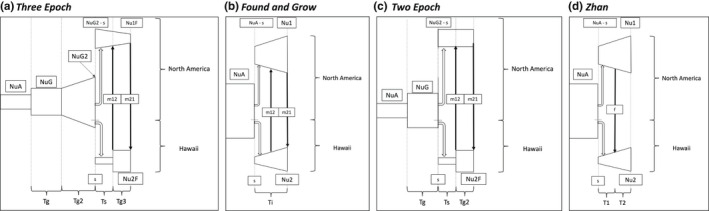
Visual depiction of the four best performing models from dadi simulations. (a) *Three epoch*, which allows for multiple changes in the size of the ancestral north American population prior to establishment in Hawaii, the north American and Hawaiian populations to change in size after the Hawaiian establishment, and a constant migration rate between north American and Hawaii after a brief time lag. (b) *Found and grow*, which assumes a constant ancestral north American population size. (c) *Two epoch*, which has a one‐time admixture rather than constant migration. (d) the model from Zhan et al. (2014), which allows for only a single instance of growth in the north American population prior to the establishment of the Hawaiian population. In each model, parameters are estimated jointly and correspond to the following: Nu = population size at a particular time, T = time, m = migration rate between populations and s = founding population size. Note that occasions of population size change depicted here are allowed to freely optimize to be either population growths or declines.

We fit a range of possible models to the observed data: (i) each of the models in the “Island Model” set described in the dadi_pipeline (Portik et al., 2017), which contains some models originally published in Charles et al. (2018); (ii) the model described by Zhan et al. (2014) for the same comparison; and (iii) a similar model that allowed for an additional period of growth prior to the establishment of the Hawaiian population and another following establishment (hereafter referred to as the *Three Epoch* model). The latter two models, as well as the two dadi_pipeline models which performed well, are shown schematically in Figure 1. To allow for a more realistic population growth trajectory, we also ran each of the dadi_pipeline models with a logistic population growth equation rather than the exponential growth variant defined in the original models. Each of the dadi_pipeline models and their logistic growth versions were run three times: once with growth allowed in the founding population post‐split, once with growth allowed in the founded population post‐split, and once with growth allowed in both populations post‐split. Note that in each of these (dadi_pipeline) models, a source population splits to form two descendant populations, with an optimized parameter (*s*) controlling the portion of the population that forms each descendant population. When *s* is optimized to be very small (as it typically was), the founded population represents only a very small proportion of the ancestral population, as is probably realistic for the founding of the Hawaiian population from the North American population. Schematic depictions of the dadi_pipeline models are available in Portik et al. (2017) and Charles et al. (2018).

To optimize the models we fit during the analysis, we used a variation of the dadi_pipeline, the sequential step‐down parameter permutation approach described by Portik et al. (2017). Unlike this method, however, we set the starting parameters for each sequential run via weighting the parameters from each run in the previous iteration by the relative Akaike information criterion (AIC) score of that iteration, such that all but the worst runs contribute in some degree to the starting parameters for the next step. The number of runs and iterations per step are listed in Table S3. Individual optimization runs were killed if they took longer than 48 h to complete; these runs tended to take far longer to finish and often included integration errors due to extremely small population sizes, resulting in extremely large amounts of genetic drift. Most runs completed in under 48 h and are included in the results.

### 2.9 Parameter estimation

To extract meaningful parameter units from the results, we assumed 0.3 years per generation and used the per‐base mutation rate of 8.4 × 10^−9^ reported from *Drosophila melanogaster* (Haag‐Liautard et al., 2007). We use these values to match those used by Zhan et al. (2014) for ease of comparison. We also used a potentially more realistic generation time of seven generations per year and the slower mutation rate reported for the more closely related *Heliconius melpomene* of 2.9 × 10^−9^ (Keightley et al., 2015). To determine the length of the considered genomic region, we multiplied the total number of bases sequenced after quality filtering (but not SNP *p*‐value filtering so as to count nonpolymorphic sites) by the ratio of SNPs in the final allele frequency spectrum to the total number of called SNPs.

## 3 RESULTS

### 3.1 Sequencing results

After paralogue filtering, we were able to genotype 2,159,978 sites in at least 50% of individuals. In total, 541,899 of these sites were polymorphic, and 71,157 had a minor allele frequency above 0.05. For data set 1, we retained 11,384 loci after removing loci within 10 kb of each other. Data sets 2 and 3 had 70,878 and 413,271 sites, respectively. The number of samples from each population after filtering is shown in Table 1. Note that some populations had no samples that passed filtering, and so are not included in Table 1.

**TABLE 1:** Number of samples remaining after filtering, Tajima's *D*, observed heterozygosity (*H*_O_) and nucleotide diversity (π).

Population	No. of samples	Tajima's *D*	*H*_O_	π
North America (NAM)	83	−1.92	0.055	0.064
Hawaii (HAW)	9	−0.211	0.048	0.055
Guam (GUA)	19	0.091	0.031	0.032
Rota (ROT)	16	0.388	0.035	0.038
Saipan (SAI)	4	0.326	0.022	0.025
Queensland (QLD)	15	0.349	0.042	0.044
New South Wales (NSW)	5	0.433	0.038	0.042
Victoria (VIC)	2	0.899	0.041	0.043

*Note*: Populations from Guam, Rota and Saipan are all part of the Mariana Islands archipelago. Samples from Maui and Oahu are pooled into a single Hawaiian population. Queensland, New South Wales and Victoria are all within the Australian continent. Populations from Samoa, New Caledonia, Fiji and New Zealand are not shown because no samples remained from these populations after filtering (but see Figure 2).

### 3.2 Overall patterns of relatedness

**FIGURE 2:**
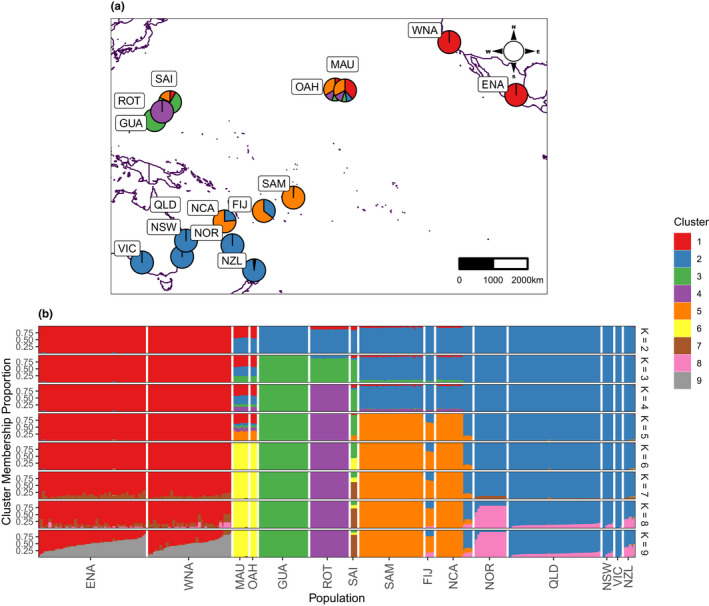
Relatedness among sampled populations. (a) Map of sampled populations, with pie charts reflecting average population results from NGSadmix (*k* = 5). Note that the geographical positions of the Mariana Islands (ROT, GUA and SAI) and the Hawaiian islands (OAH and MAU) are shifted slightly for readability. (b) NGSadmix plots showing the proportion of ancestry across clustering values between *k* = 2 and *k* = 9. At *k* = 5, Hawaii reflects a mixture of ancestry comprising north American, Mariana Islands and southwestern Pacific samples. At *k* = 6, Hawaii becomes its own cluster. At values beyond *k* = 6, populations are subdivided.

PCA separated North American, Hawaiian, Mariana Islands and southwest Pacific samples along two axes of expansion (Figure S1B). NGSadmix results showed a similar result (Figure 2b), splitting the North American samples from the Pacific samples at *K* = 2, the Mariana Islands at *K* = 3, Rota from the other Mariana Islands at *K* = 4, Samoa, Fiji and New Caledonia at *K* = 5, Hawaii at *K* = 6, Saipan from Guam at *K* = 7, and Norfolk Island at *K* = 8. Notably, the Hawaiian samples were consistently classed as having ancestry partially from all other clusters until *K* = 6, consistent with an initial introduction into the archipelago. At *K* = 9, nearly all North American samples were assigned to two genetic clusters with ancestry proportions unrelated to their geographical sampling locations, which can be interpreted as the presence of a fictive cluster with no biological significance (e.g., Chen et al., 2007; Guillot et al., 2005). *K* = 2 and *K* = 5 had the highest Δ*K* values (Evanno et al., 2005), although we were not able to estimate Δ*K* for *K* = 3 due to very low likelihood variance between runs at *K* = 2 and *K* = 3, thereby producing an undefined Δ*K* (Figure S2). Eastern and Western North America were never split. Directionality index (*ψ*) scores indicated westward establishments (Figure S4). Genetic diversity (π, *H*_O_ and Het/Hom) was highest in the ancestral North American populations, followed by Hawaii, Australia and then the remaining Pacific Island populations (Table 1; Figure S4). Tajima's *D* was positive in all sites except North America and Hawaii (Table 1). *F*_ST_ results also reflect the patterns we observed in the PCA and NGSadmix results (Figure 2b; Table S2, Figure S1A). These results varied slightly in the heavily filtered data set, but followed the same general trends (with a higher diversity in North America, Hawaii and Australia, and very similar Tajima's *D* and *F*_ST_ results, Tables S5 and S6). These results did have a higher relative diversity in the Pacific populations than in the full data set, however, and, as a natural consequence of removing loci with low minor allele frequencies, much higher overall diversity estimates.

### 3.3 Patterns of differentiation within expansion populations

Samples from the Mariana Islands (especially the well‐sampled Guam and Rota populations) appear to form highly distinct populations, despite their close physical proximity (Figure 2a,b; Figure S1A,B). By contrast, populations within Hawaii (Maui and Oahu) and Australia (Queensland, New South Wales and Victoria) do not show strong patterns of differentiation (Figure 2a,b). Norfolk Island, the other previously unsampled population in our data set, groups closely with samples from Australia and New Zealand (Figure 2a,b; Figure S1A,B). IBD patterns were significant within the Mariana Island (*p* = .001, *r* = .723), North American (*p* = .001, *r* = .036) and Hawaiian (*p* = .005, *r* = .456) samples, and were present but not significant within Australian samples (*p* = .109, *r* = .122). In the heavily filtered data set, IBD patterns were significant within all except Hawaii (*p* = .001, *r* = .593 in the Mariana Islands; *p* = .002, *r* = .054 in North America; *p* = .609, *r* = .131 in Hawaii; and *p* = .014, *r* = .238 in Australia).

### 3.4 Timing of establishment and patterns of ongoing gene flow

Among the large set of possible demographic models, the simple *Found and Grow* scenario (Figure 1b), which had a constant ancestral population size in North America, Hawaii colonization and then population growth in both sites, produced the lowest AIC scores on the final pass of the pipeline (Figure S5). However, the *Two Epoch* model, which had a single admixture event but no consistent migration (Figure 1c), had the lowest AIC score across all passes of the pipeline. The new *Three Epoch* model (Figure 1a), which involved multiple rounds of demographic expansion in the ancestral North American population, followed by colonization and growth in Hawaii, had a lower AIC score than *Found and Grow* across all passes and a lower AIC score than the *Two Epoch* model on the final pass (Figure S5). These were the top three models across all passes and in the final pass alone (Figure S5, Table S4). The *Three Epoch* model is a more complex version of the model specified in Zhan et al. (2014, hereafter *Zhan*). We therefore report only the results for the *Found and Grow*, *Three Epoch*, *Two Epoch* and *Zhan* models here.

**FIGURE 3:**
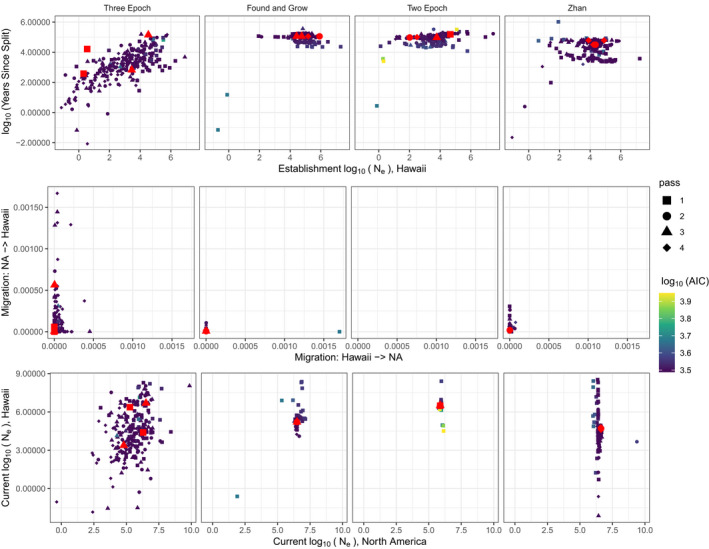
Results of the dadi optimization runs for the (left to right) *three epoch*, *found and grow*, *two epoch* and *Zhan* demographic models. In the key at right, “pass” refers to each iteration of the sequential step‐down permutation approach (see methods: dadi model selection), and the log_10_(AIC) score corresponds to the likelihood of observing a particular set of parameter values. (top row) estimated effective size of the founding population in Hawaii vs. years since establishment. (middle row) estimated migration rates from North America to Hawaii and vice versa. (bottom row) estimated current effective population sizes in North America and Hawaii. Red dots mark the runs with the lowest AIC scores in each quadrant of the respective parameter space; these runs correspond to the heatmaps in Figure 4 and Figures S6 and S7 and the residuals in Figure S8. Note that the *two epoch* model does not include a constant migration rate between Hawaii and North America, and so is blank for the middle panel.

**FIGURE 4:**
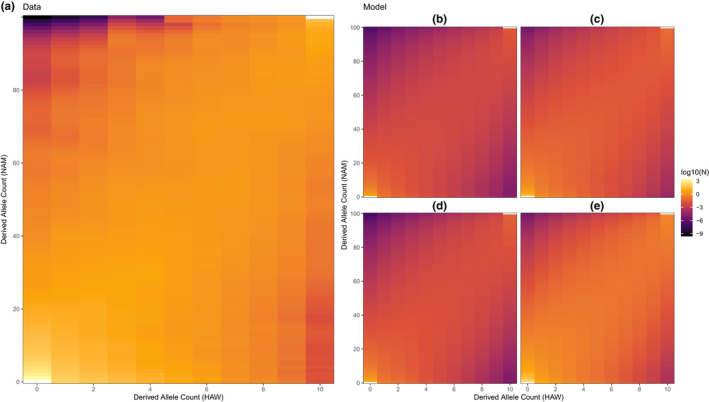
(a) observed data and (b‐e) model‐estimated derived site frequency spectra for the *three epoch* model. Cell brightness corresponds to the number of loci with derived allele frequencies in the given bin for both Hawaii (HAW) and North America (NAM). Note that spectra have been projected to a size of 100 for North America and 10 for Hawaii to match the approximate number of individuals sequenced from each population. Model‐derived site frequency spectra correspond to the four points marked in red in Figure 3 for the *three epoch* model, which are parameter estimates from the runs with the lowest AIC score. Figures S6–S8 show comparable plots for the other candidate demographic models. BL, bottom left; BR, bottom right; TL, top left; TR, top right.

These four models gave highly variable estimates of establishment timing and founding population size, with the *Three Epoch* model generally producing much broader estimates for these parameters. For example, while the *Three Epoch* model suggested establishment times that ranged between ~10^2^ and 10^5^ years ago, the other models suggested times between 10^4^ and 10^5^ years ago (Figure 3). Similarly, the latter models were more consistent in predicting a large founding population of 10^3^–10^6^ individuals, while the *Three Epoch* models suggested a broader founding population size of between 10 and 10^6^ individuals (Figure 3). These models also differed in their estimates of the contemporary *N*_*e*_ for the Hawaiian population, with the *Found and Grow* and *Two Epoch* models suggesting a large *N*_*e*_ of around 10^6^ and the *Three Epoch* and *Zhan* models generally producing estimates of Hawaiian *N*_*e*_ between 10^2^ and 10^7^ (Figure 3). For all of the models, the major discrepancy between the observed and simulated SFS tended to be that the models underestimated the number of rare derived alleles in the North American but not the Hawaiian populations (Figure 4; Figures S6–S9). This pattern may indicate that the models did not optimize for strong enough founder effects in Hawaii, which would have caused a more drastic loss of rare alleles.

For other parameters, the four models generated similar estimates. Each of these models suggest very low levels of contemporary migration between North America and Hawaii, with the *Found and Grow* and *Two Epoch* models converging near 0 for both directions and the *Three Epoch* model generally suggesting migration rates of <5 × 10^−4^ and <2.5 × 10^−4^ for individuals per generation from North America to Hawaii and from Hawaii to North America, respectively, and the *Zhan* model suggesting migration rates of <2.5 × 10^−4^ in either direction (Figure 3). Using a more accurate generation time and mutation rate than the values used by Zhan et al. (2014) produced a result with slightly more distant divergence times and larger effective sizes, but not to a substantial degree.

## 4 DISCUSSION

Many geographical range expansions occur via serial stepwise dispersal, and we found strong evidence for this pattern in Pacific monarch butterflies, consistent with a previous study (Pierce, Zalucki, et al., 2014a). Monarchs in the Mariana Islands are the product of a distinct expansion event within the Pacific. Summary statistics support a scenario of directional dispersal from North America to Hawaii, from Hawaii to Guam, and from Hawaii to Australia. This pattern is reflected in both the positive directionality index measures (0.07, 0.08 and 0.05, respectively) (Peter & Slatkin, 2013) and other summary statistics, such as the general increase in Tajima's *D* across the Pacific, which is consistent with stronger or more recent population bottlenecks during successive colonization. Interestingly, monarch populations in Hawaii and Australia seem to maintain relatively high levels of genetic diversity, despite the apparent bottlenecks associated with establishment. This is especially striking in the Australian population, which was itself probably founded by individuals from a much smaller population in New Caledonia (Clarke & Zalucki, 2004). The retention of genetic diversity in Hawaii and Australia may reflect rapid population growth upon establishment, which could temper the loss of allelic diversity that might be predicted with a bottleneck event, akin to the scenario described in Hawaiian *Drosophila* by Nei et al. (1975). The slightly negative Tajima's *D* value in Hawaii is consistent with population growth following a bottleneck and is consistent with this hypothesis.

Within the Mariana Islands, there was a strong pattern of differentiation between islands, especially between the nearby islands of Guam and Rota. This pattern is striking because of their close geographical proximity: these islands are separated by only 40 km of open ocean. By contrast, our samples from North America, despite coming from overwintering sites nearly 2000 km apart, formed a single genetically indistinguishable population. This pattern is apparent from the strong pattern of IBD observed within Mariana Islands samples compared to weak/no IBD within North America. Our results are similar to those of Dapporto et al. (2017) and Vodă et al. (2016), who also noted strong genetic differentiation between butterfly lineages even from nearby islands, as well as Alvial et al. (2018), who showed that a migratory dragonfly exhibits little genetic differentiation across its migratory Central and South American range but substantial genetic differentiation between nonmigratory populations on Pacific islands. However, our findings are unique because the observed differences in population structure between migratory and nonmigratory populations developed very recently, probably emerging over the past 150 years.

The lack of differentiation within North American monarchs corroborates other population genetic analyses of eastern and western North American monarchs (Brower & Boyce, 1991; Lyons et al., 2012; Shephard et al., 2002; Talla et al., 2020; Zhan et al., 2014) and is consistent with studies that have suggested movement of individuals between eastern and western North America (Billings, 2019; Dingle et al., 2005; Morris et al., 2015). The strong population genetic differentiation within the Mariana Islands but not at the scale of the entire North American continent highlights both (i) the pervasive role that long‐distance migration in North America plays in collapsing any patterns of population structure that might otherwise develop, and (ii) the fact that many nonmigratory Pacific monarch populations probably have extremely small effective population sizes that are susceptible to very strong genetic drift.

In contrast to populations within the Mariana Islands, Hawaiian and Australian monarchs show only modest evidence for IBD that might be expected in nonmigratory monarch populations. Within Hawaii, our samples from Maui and Oahu formed a single genetic cluster, consistent with the results of Pierce, de Roode, et al. (2014b). Likewise, Australian samples from New South Wales and Victoria grouped with samples from Queensland. This result differs somewhat from the results of Hughes and Zalucki (1984), who reported considerable among‐site genetic variation within Queensland, but it is consistent with similar later work (Zalucki et al., 1987).

For Hawaiian monarchs, it is not immediately clear why the islands of Maui and Oahu do not form clearly distinct populations. One possibility is that prevailing winds promote gene flow between islands in a way that differs from the Mariana Islands. Pacific monarchs are likely to be moved by wind patterns, similar to wind‐driven movement patterns noted in migratory *Vanessa cardui* (Stefanescu et al., 2007), and it has been suggested that a tropical cyclone may have led to the monarch's establishment in Australia, following an “outbreak” of monarchs shortly after establishing in New Caledonia (Clarke & Zalucki, 2004). Another possibility is between‐island movement of monarchs by butterfly breeders in Hawaii, who sell monarchs for release at weddings and celebrations (D. Loo‐McDowell, pers. comm.). In the case of Australian monarchs, the lack of strong differentiation across the continent may be driven by seasonal migration patterns akin to those seen in western North American monarchs (James, 1993; James & James, 2019). Australian monarchs retain migration‐associated behaviours such as seasonal reproductive arrest and sustained directional flight—necessary although not sufficient conditions for long‐distance migration—that further support the notion that they may undergo large‐scale seasonal movements (Freedman et al., 2018; W. B. Hemstrom, M. P. Zalucki, M. G. Freedman, & M.R. Miller, in prep.; James, 1993). Thus, the lack of continent‐wide population structure seen in migratory North American monarchs may be recapitulated, albeit to a lesser extent, in Australia.

Interpreting the results of our demographic models is somewhat more complicated than interpreting basic patterns of relatedness among populations. This is due to the conflicting inferences provided by the two best‐performing model structures and the wide range of parameter estimates in the *Three Epoch* models. Although we present the results of both the simpler *Found and Grow* and *Two Epoch* models, and the more complicated *Three Epoch* model, we are inclined to place more confidence in the estimates produced by the *Three Epoch* model for two reasons: (i) the demographic scenario that it specifies—recent demographic expansion in the ancestral North American population prior to geographical expansion—has empirical support from other studies (Boyle et al., 2022; Pfeiler et al., 2017; Zhan et al., 2014) and accords with our understanding of past changes in climate, and (ii) this model structure produces parameter estimates that match our prior understanding for how and when monarch range expansion may have occurred. The latter point is related to the former: because a North American population expansion is not allowed until after the founding of Hawaii in the *Found and Grow* model, this model forces an ancient founding of the Hawaiian population in order to allow for the ancient growth of the North American population. As such, we focus our discussion on the estimates produced by the *Three Epoch* model.

In general, our demographic results do not exclude a recent founding of the Hawaiian population by North American monarchs (Figure 3d). While our model optimizations span several orders of magnitude for the time since establishment, many of the iterations settled on introduction estimates of less than 200 years ago for the *Three Epoch* model. Since the earliest historical records of monarchs on Hawaii date to roughly 200 years ago (1841) (Zalucki & Clarke, 2004), we are inclined to accept the results of iterations with shorter estimated divergence times. Other lines of evidence supporting recent (<200 years) Hawaiian establishment include: (i) the lack of noticeable phenotypic differentiation between North American and Pacific Island monarchs, especially relative to the pronounced phenotypic differences in nonmigratory populations from the Caribbean and South America (Freedman et al., 2020), which have historically been treated as separate subspecies (Ackery & Vane‐Wright, 1984); (ii) the probable need for human‐mediated transport of the monarch's host plants (some of which are native to subtropical Africa) as a precondition of monarch establishment in the Pacific; and (iii) recent genomic evidence showing that captive breeding of monarchs over short timescales is sufficient to generate patterns of genetic divergence comparable to those observed between North American and Pacific populations (Tenger‐Trolander et al., 2019). Notably, our re‐implementation of the model used by Zhan et al. (2014) produced results similar to theirs, with the majority of model iterations supporting an introduction time >1000 years ago (Figure 3). This highlights the need to run a range of possible demographic models when attempting to infer demographic history, since failing to account for underlying complexity in population histories can result in very divergent parameter estimates.

One complication for interpreting our demographic models is that they consistently underestimated the number of rare, derived alleles present in North America but not Hawaii. During very strong bottlenecks, we would expect many rare alleles to be lost, suggesting that our models may be overestimating the founding population size, and thus probable establishment date. Since dadi can struggle to calculate SFS when population sizes are very small due to large amounts of drift, iterations that optimize to this segment of parameter space are more likely to have integration errors or very long processing times. These uncompleted runs were not included in our model results, and so this part of the parameter space may be inadequately explored. Since small founding population sizes correlated with recent introductions across our model results, this also suggests that it would be unwise to rule out a recent introduction with a very strong bottleneck based on a demographic analysis alone.

Demographic model results were also variable in their estimates of founding population sizes in Hawaii. Some models produced estimates as high as 10,000 founding individuals, which seems implausible given the long distance (>3500 km) between North America and Hawaii. The extremely wide range of parameter estimates for founding population size and timing may reflect that, in practice, it is difficult to distinguish between a very recent strong bottleneck vs. a more distant but less severe bottleneck. Our model results are consistent with this, since the model iterations with recent establishments also tended to have smaller establishment population sizes (Figure 3).

In contrast to variable estimates of establishment timing and founding population size, demographic models were consistent in suggesting very low contemporary migration rates (on the order of 0.0001 individuals per generation from North America to Hawaii and vice versa). Our results thus contrast with those of Pierce, Zalucki et al. (2014a), who inferred much higher migration rates (nearly 10 individuals/generations) between North America and Hawaii. We are more confident in our results due to (i) the much larger number of sampled loci, (ii) the more realistic demographic model that we used in our analysis and (iii) the absence of modern records of regular North America to Hawaii establishment events. However, we do note that monarchs appear to be capable of wind‐aided dispersal over extremely long distances, as evidenced by occasional records from the UK that coincide with records of migratory North American birds blown by storms (Asher et al., 2001); thus, ongoing movement from North America to Hawaii is not entirely implausible.

Understanding how migratory and nonmigratory populations of monarchs differ genetically, phenotypically and ecologically has important conservation implications. First, monarchs were recently evaluated by the U.S. Fish and Wildlife Service, who determined that a listing under the U.S. Endangered Species Act is “warranted but precluded” (USFWS, 2021). This decision will be re‐evaluated annually, and the retention of genetic diversity in nonmigratory monarch populations from outlying US states/territories (Hawaii, American Samoa, the Mariana Islands, Puerto Rico, US Virgin Islands) may be important in decisions regarding the adaptive capacity of the species (Freedman et al., 2021). Second, some recent evidence suggests that climate warming and planting of nonnative milkweed species might tip the scales in favour of year‐round breeding and loss of migratory behaviour in North American monarchs, particularly in western North America (Crone & Schultz, 2021; James, 2021; James et al., 2021). The increased prevalence of partial migration within North America, both along the US Gulf Coast and in California (James et al., 2021; Satterfield et al., 2016, 2018), may affect patterns of spatial genetic diversity: for example, if future sequencing of North American monarchs finds evidence for population structure within areas of their range where year‐round breeding occurs, this would provide evidence that loss of migration is actively driving genetic differentiation. Finally, our results are also helpful for monarch conservation because they provide a relatively large sample of North American monarchs (*n* = 90) against which future sequencing efforts can be compared to look for evidence of contemporary losses of genetic diversity associated with potential population decline.

## AUTHOR CONTRIBUTIONS

WBH, MGF and MRM designed the research. MGF and MPZ provided samples used for sequencing. WBH and MGF performed data analysis. All authors contributed to writing and editing the manuscript.

## Conflict of Interest

The authors declare no conflicts of interest related to this work.

## FUNDING INFORMATION

W.B.H. was funded through the University of California, Davis Animal Science Department. M.G.F. received funding through the NSF Graduate Research Fellowship, the National Geographic Society, the Society for the Study of Evolution, and the NSF EAPSI programme. Funding for sequencing was provided by the University of California, Davis Animal Science Department to M.R.M. M.P.Z. was supported by the University of Queensland, and S.R.R. was supported by the David and Lucile Packard Foundation.

### OPEN RESEARCH BADGES

“This article has earned an Open Research Badge for making publicly available the data and scripts necessary to recreate the analyses and results reported in this manuscript. All of these resources are available at https://github.com/hemstrow/F‐H_2018.”

## Supporting information


Appendix S1

## Data Availability

All scripts and metadata used in analyses are available at https://github.com/hemstrow/F‐H_2018. Sequence data have been deposited to the NCBI Sequence Read Archive under BioProject ID PRJNA848092.
