# Supplementary material for: Population genetics of a recent range expansion and subsequent loss of migration in monarch butterflies
Source: Mol Ecol. 2022 Jul 21;31(17):4544–57. doi: 10.1111/mec.16592 (PMC9546011; doi:10.1111/mec.16592)
Supplement: Supplementary file 1 — Appendix S1 [file MEC-31-4544-s001.docx]

**Supplementary Methods**

*Filtered Datasets*

In addition to datasets 1-4 described in the main text, we also used a few more heavily filtered datasets for comparison.

1. In order to determine if our filtering approach influenced our diversity results from dataset 2, we also used a dataset produced using the 10kb gapped SNP dataset (dataset 1) used for the demographic analysis after removing all loci with a p-value for Hardy-Weinberg Equilibrium (HWE) below 1 x 10^-6^ in any individual population or with a minor allele frequency below 0.05 in every population. HWE was calculated according to Wigginton et al. (2005). This dataset was used to re-run all of the analyses that depended on dataset 2.
2. To likewise determine if our Tajima’s D results from dataset 3 were influenced by our lack of filtering, we also created a slightly edited version of dataset 3 to include the same HWE filter as in dataset 4.

For dataset 5, we removed 362 SNPs that violated our minor allele frequency filter and 86 SNPs that violated our HWE filter, resulting in 10,707 loci after removing poorly sequenced individuals. The number of samples that were retained in each population are shown in Table S4.

*Demographic analysis*

To optimize the models we fit during the analysis, we used a variation on dadi_pipeline, the sequential step-down parameter permutation approach described by Portik et al. (2017) [1]. Unlike this method however, we set the starting parameters for each sequential run via weighting the parameters from each run in the previous iteration by the relative AIC score of that iteration, such that all but the worst runs contribute in some degree to the starting parameters for the next step. The number of runs and iterations per step are listed in Table S3. Individual optimization runs were killed if they took longer than 48hrs to complete, since these runs tended to take far longer to finish and often included integration errors due to extremely small population sizes. Most runs completed in under 48hrs and are included in the results, save for the founder_asym_growth_pop_2 model, for which only a handful completed due (likely due to very small optimal population sizes in Hawaii). The number of runs completed for each model as well as the minimum, mean, and the standard deviation of both the AIC and log(likelihood) scores for each model at each step are shown in Table S4. Graphic depictions for each of the models for that for which one was not included in the main text are available from the dadi_pipeline GitHub repository (https://github.com/dportik/dadi_pipeline/blob/master/Two_Population_Pipeline/Models_2D.pdf). The models represent those depicted here as of August 13^th^, 2021.

**Supplementary References**

1. Portik DM, Leaché AD, Rivera D, Barej MF, Burger M, Hirschfeld M, Rödel M-O, Blackburn DC, Fujita MK. 2017 Evaluating mechanisms of diversification in a Guineo-Congolian tropical forest frog using demographic model selection. *Mol. Ecol.* **26**, 5245–5263. (doi:10.1111/mec.14266)
2. Haag-Liautard C, Dorris M, Maside X, Macaskill S, Halligan DL, Charlesworth B, Keightley PD. 2007 Direct estimation of per nucleotide and genomic deleterious mutation rates in Drosophila. *Nature* **445**, 82–85. (doi:10.1038/nature05388)
3. Zhan S *et al.* 2014 The genetics of monarch butterfly migration and warning colouration. *Nature* **514**, 317.
4. Keightley PD, Pinharanda A, Ness RW, Simpson F, Dasmahapatra KK, Mallet J, Davey JW, Jiggins CD. 2015 Estimation of the spontaneous mutation rate in Heliconius melpomene. *Mol. Biol. Evol.* **32**, 239–243. (doi:10.1093/molbev/msu302)

**Supplementary Tables:**

| **Population** | **Sampling Location** | **Sampling Year(s)** | **# Sequenced** |
| --- | --- | --- | --- |
| **North America** | Eastern North America (Mexican overwintering sites) | 2016 | 45 |
|  | Western North American (various California overwintering sites) | 2015 | 40 |
| **Hawaii** | Maui (Kapalua, Kihei) | 2016 | 8 |
|  | Oahu (Honolulu) | 2016 | 4 |
| **Mariana Islands** | Guam (near Dededo) | 2015 | 24 |
|  | Rota (near Songsong) | 2015 | 20 |
|  | Saipan (Kagman) | 2015 | 4 |
| **Fiji** | Viti Levu | 2009 | 5 |
| **Samoa** | Upolu | 2006, 2007, 2016 | 31 |
| **New Caledonia** | Grand Terre | 1991, 2006, 2010 | 18 |
| **Australia** | Queensland (Samford, Canungra, Pinjarra Hills) | 2016 | 44 |
|  | Victoria (near Melbourne) | 2016 | 4 |
|  | New South Wales (Wisemans Ferry) | 2016 | 6 |
| **New Zealand** | North Island | 2007, 2011 | 6 |
| **Norfolk Island** | Norfolk Island | 2016 | 16 |
| **Total:** | | | **275** |
| **Table S1** – Sampling locations and year for monarchs included in sequencing design. | | | |

| **Population** | **GUA** | **HAW** | **NAM** | **NSW** | **QLD** | **ROT** | **SAI** | **VIC** |
| --- | --- | --- | --- | --- | --- | --- | --- | --- |
| **GUA** |  | 0.2649 | 0.264 | 0.3546 | 0.3258 | 0.3018 | 0.3039 | 0.3885 |
| **HAW** | 0.001 |  | 0.1 | 0.1288 | 0.1426 | 0.1985 | 0.2411 | 0.1083 |
| **NAM** | 0.001 | 0.001 |  | 0.1824 | 0.1874 | 0.2218 | 0.2703 | 0.1758 |
| **NSW** | 0.001 | 0.002 | 0.001 |  | 0.011 | 0.2862 | 0.3698 | -0.0393 |
| **QLD** | 0.001 | 0.001 | 0.001 | 0.2248 |  | 0.2702 | 0.3366 | 0.0186 |
| **ROT** | 0.001 | 0.001 | 0.001 | 0.001 | 0.001 |  | 0.3627 | 0.3024 |
| **SAI** | 0.001 | 0.001 | 0.001 | 0.001 | 0.001 | 0.001 |  | 0.4227 |
| **VIC** | 0.001 | 0.028 | 0.003 | 0.8112 | 0.1708 | 0.001 | 0.001 |  |
| **Table S2 -** Average pairwise F_ST_ across all sites for each pair of populations. F_ST_ above the diagonal, p-value from 1,000 permutations below. | | | | | | | | |

| **Run** | **Parameter Permutation** | **Number of Runs** | **Number of Optimization Iterations** |
| --- | --- | --- | --- |
| **1** | 3 | 100 | 30 |
| **2** | 2 | 50 | 50 |
| **3** | 2 | 60 | 50 |
| **4** | 1 | 100 | 100 |
| **Table S3 –** Degree of parameter permutation, number of independent dadi runs, and the number of iterations per run for each of the dadi optimization passes (see Portik et al., 2017). | | | |

**Table S4 –** Number of completed runs as well as the minimum, mean, and standard deviations of AIC and log(likelihood) scores for each model included in the analysis. **Note: Large table, in attached excel file.**

| **Population** | **# Samples** | **Tajima's D** | **H_O_** | **π** | **Het/Hom** |
| --- | --- | --- | --- | --- | --- |
| **North America (NAM)** | 76 | -1.981 | 0.102 | 0.115 | 0.114 |
| **Hawaii (HAW)** | 5 | -0.265 | 0.105 | 0.114 | 0.117 |
| **Guam (GUA)** | 8 | 0.05 | 0.082 | 0.08 | 0.09 |
| **Rota (ROT)** | 6 | 0.371 | 0.089 | 0.091 | 0.098 |
| **Saipan (SAI)** | 4 | 0.299 | 0.062 | 0.066 | 0.066 |
| **Queensland (QLD)** | 10 | 0.308 | 0.11 | 0.111 | 0.124 |
| **New South Wales (NSW)** | 4 | 0.425 | 0.103 | 0.107 | 0.115 |
| **Victoria (VIC)** | 1 | 0.886 | 0.104 | 0.104 | 0.117 |
| **Table S5 –** Number of samples remaining after filtering, Tajima’s D, Observed Heterozygosity (H_O_), nucleotide diversity (π), and the average ratio of Heterozygous to Homozygous sites across all individuals in each population based on the more stringently filtered SNP dataset. | | | | | |

| **Population** | **GUA** | **HAW** | **NAM** | **NSW** | **QLD** | **ROT** | **SAI** | **VIC** |
| --- | --- | --- | --- | --- | --- | --- | --- | --- |
| **GUA** | #N/A | 0.2545 | 0.2537 | 0.3236 | 0.2799 | 0.2692 | 0.2608 | 0.384 |
| **HAW** | 0.001 | #N/A | 0.1028 | 0.1376 | 0.1324 | 0.1972 | 0.2601 | 0.123 |
| **NAM** | 0.001 | 0.001 | #N/A | 0.1973 | 0.1851 | 0.2129 | 0.2579 | 0.1765 |
| **NSW** | 0.001 | 0.005 | 0.001 | #N/A | 0.0125 | 0.2692 | 0.3489 | 0.024 |
| **QLD** | 0.001 | 0.002 | 0.001 | 0.2188 | #N/A | 0.2354 | 0.2941 | 0.0087 |
| **ROT** | 0.001 | 0.001 | 0.001 | 0.001 | 0.001 | #N/A | 0.3358 | 0.3015 |
| **SAI** | 0.001 | 0.001 | 0.001 | 0.001 | 0.001 | 0.001 | #N/A | 0.4492 |
| **VIC** | 0.001 | 0.0789 | 0.001 | 0.2937 | 0.2967 | 0.001 | 0.001 | #N/A |
| **Table S6 -** Average pairwise F_ST_ across all sites for each pair of populations based on the more stringently filtered SNP dataset. F_ST_ above the diagonal, p-value from 1,000 permutations below. | | | | | | | | |

**Supplementary Figures**


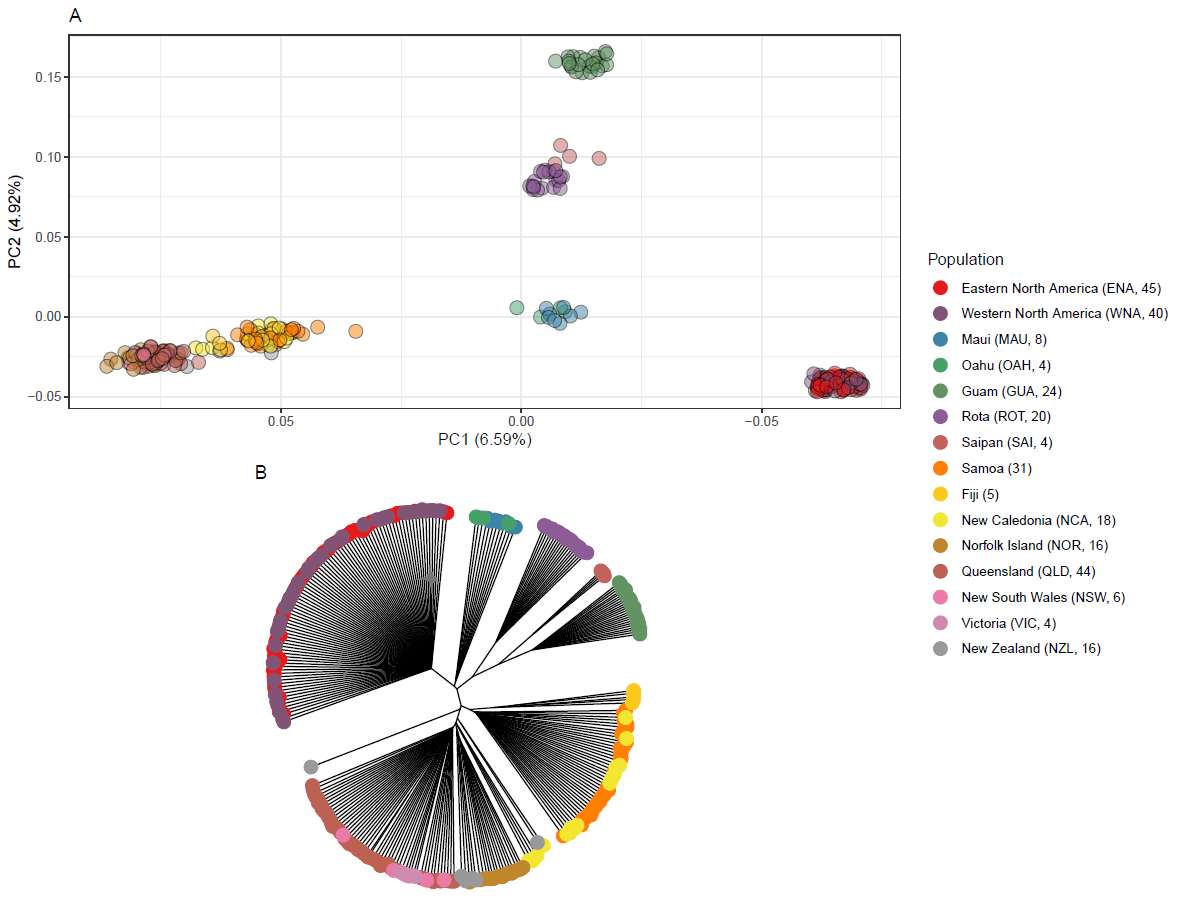


**Figure S1** – (A) PCA and (B) neighbor-joining tree depicting the relatedness amongst Monarch samples from the different sampling locations in North America and the Pacific. Note that points in (A) are slightly jittered by a factor of 0.005 to limit degree of overlap between points. Figure legend shows abbreviations used throughout manuscript for each population and the number of sample individuals per population. Note that western North American samples consistently cluster with eastern North American samples and that this shade of purple is distinct from that in Rota, which groups with other samples from the Mariana Islands (Guam and Saipan).


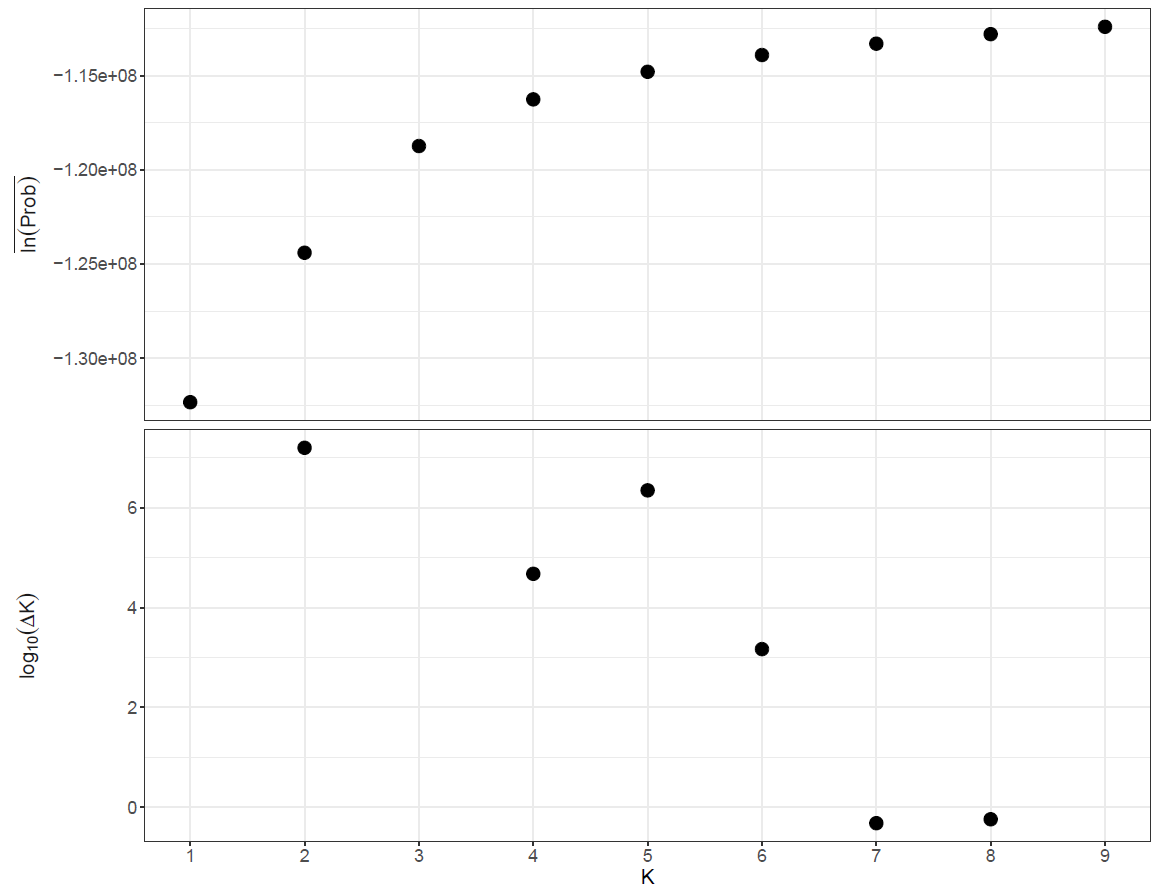


**Figure S2 –** **(top)** Mean ln(Prob) and ΔK (**bottom**) values for NGSadmix runs for each K between 1 and 9.


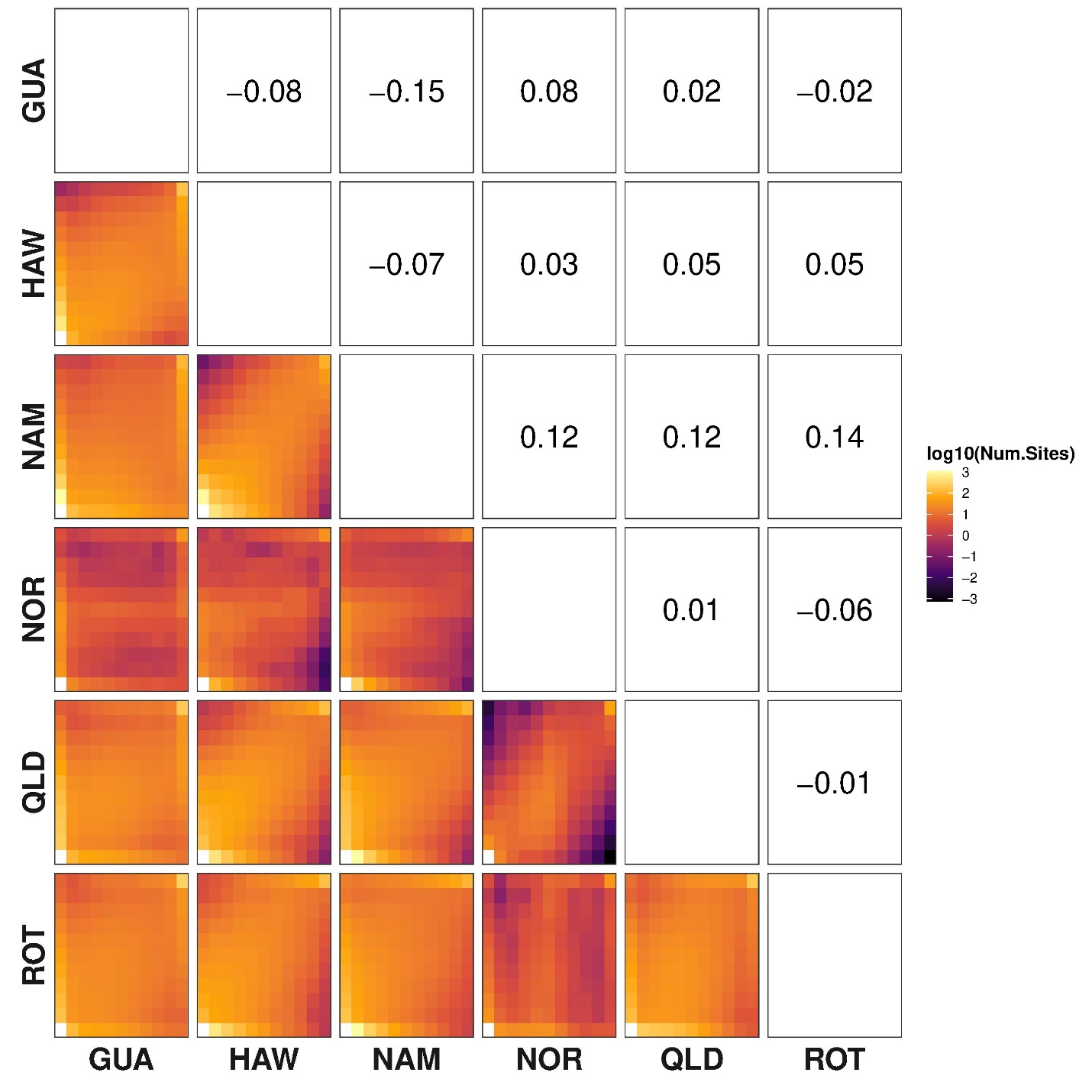


**Figure S3 –** Derived allele frequency spectra (below diagonal) and directionality indices (above diagonal) for each pairwise comparison between each of the six best-sampled populations. Spectra polarized via reference to putative sister taxon *Danaus erippus* and projected to 10 gene copies per population. GUA = Guam, HAW = Hawaii, NAM = North America, NOR = Norfolk Island, QLD = Queensland, ROT = Rota.


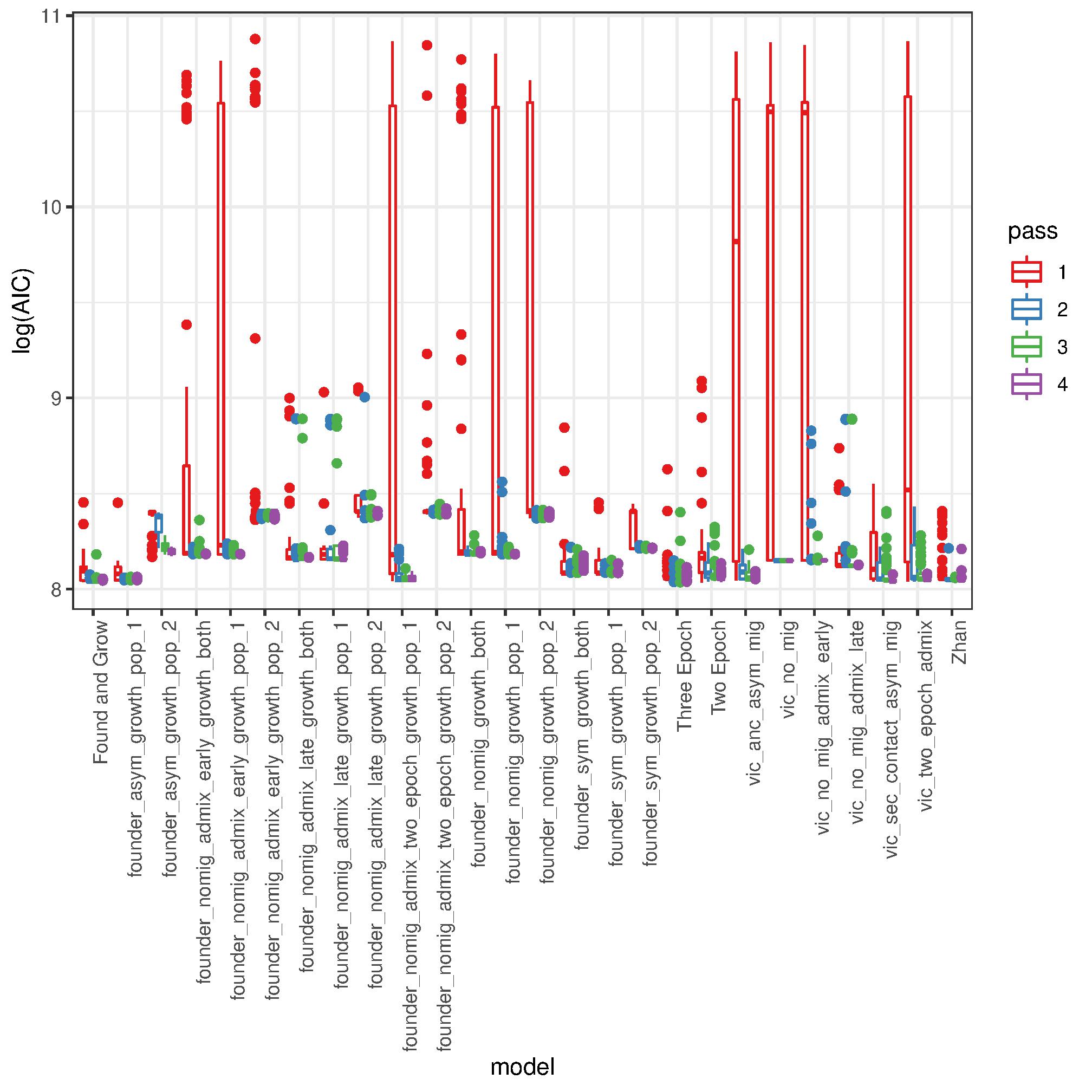


**Figure S4 -** AIC scores for all specified demographic models used in dadi simulations across passes. Note that the three epoch model produced model runs with the lowest single AIC scores, though with substantial variation across runs.


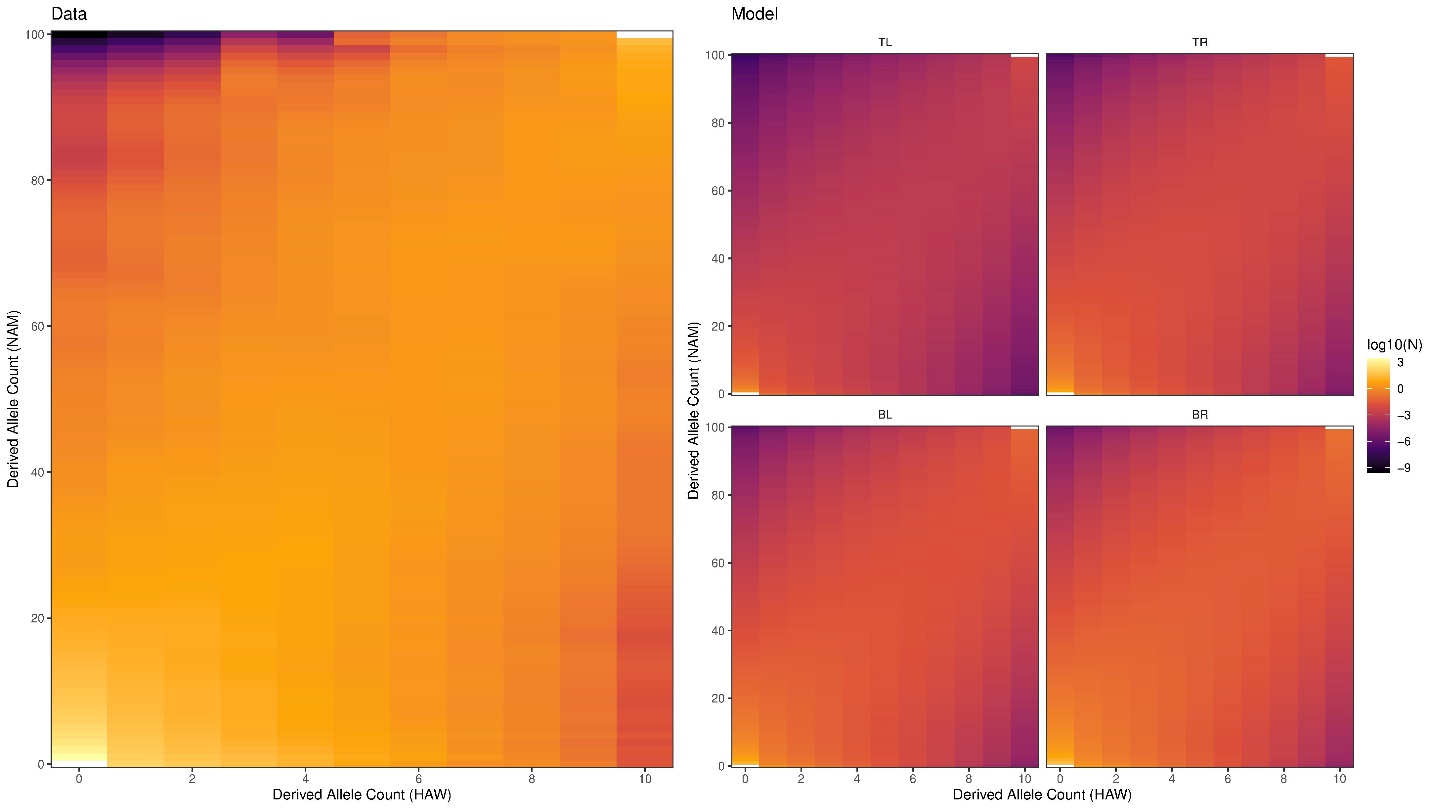


**Figure S5 –** Observed (Left) and estimated (Right) derived site frequency spectra for the *Found and Grow* model. Cell brightness corresponds to the number of loci with derived allele frequencies in the given bin for both Hawaii (HAW) and North America (NAM). Estimated spectra based on the parameters from the runs with the lowest AIC score from each quadrant of the establishment time/founding population size parameter space for each model are shown for comparison (BL: bottom left, BR: bottom right, TL: top left, TR: top right), corresponding to the points marked in red in Figure 3.


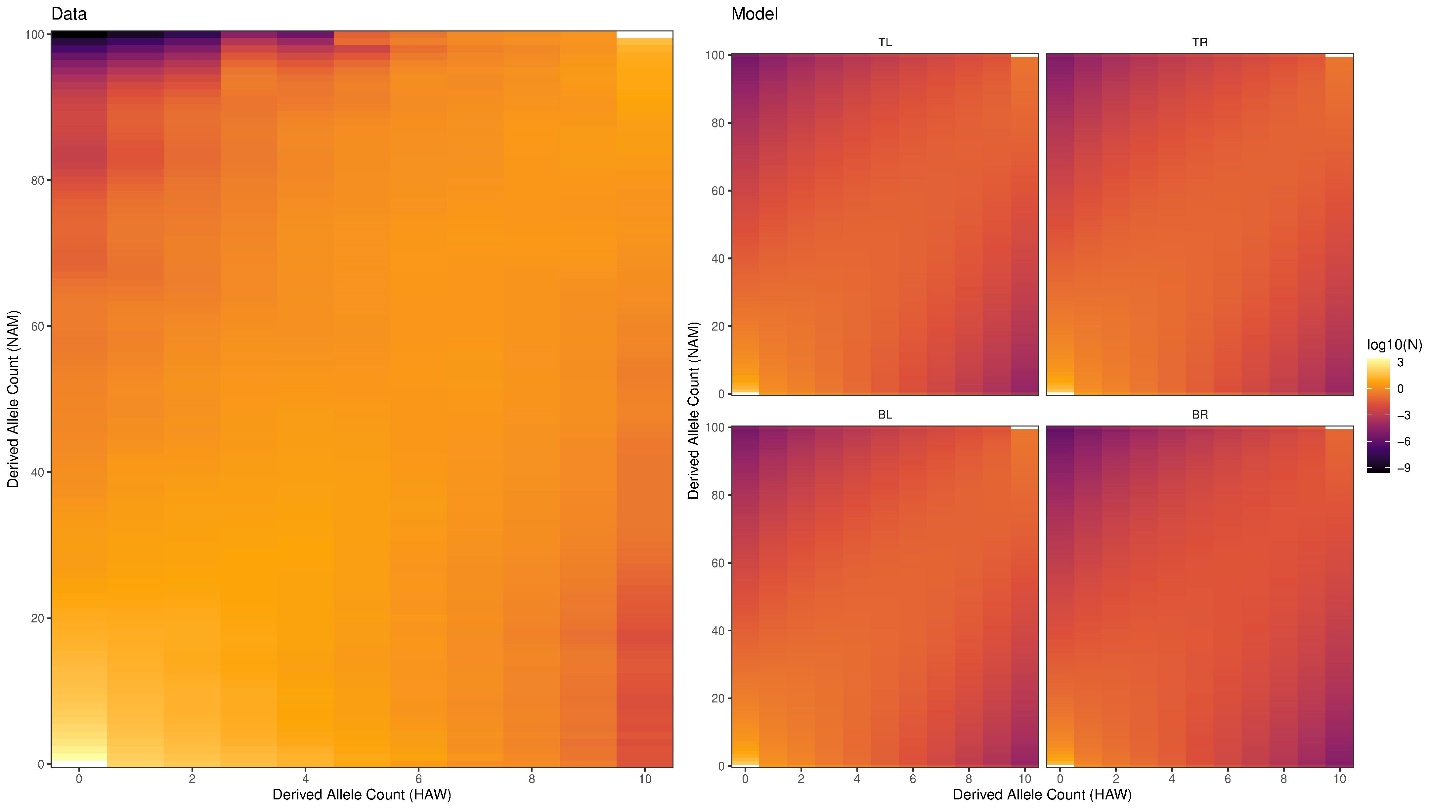


**Figure S6–** Observed (Left) and estimated (Right) derived site frequency spectra for the *Two Epoch* model. Cell brightness corresponds to the number of loci with derived allele frequencies in the given bin for both Hawaii (HAW) and North America (NAM). Estimated spectra based on the parameters from the runs with the lowest AIC score from each quadrant of the establishment time/founding population size parameter space for each model are shown for comparison (BL: bottom left, BR: bottom right, TL: top left, TR: top right), corresponding to the points marked in red in Figure 3.


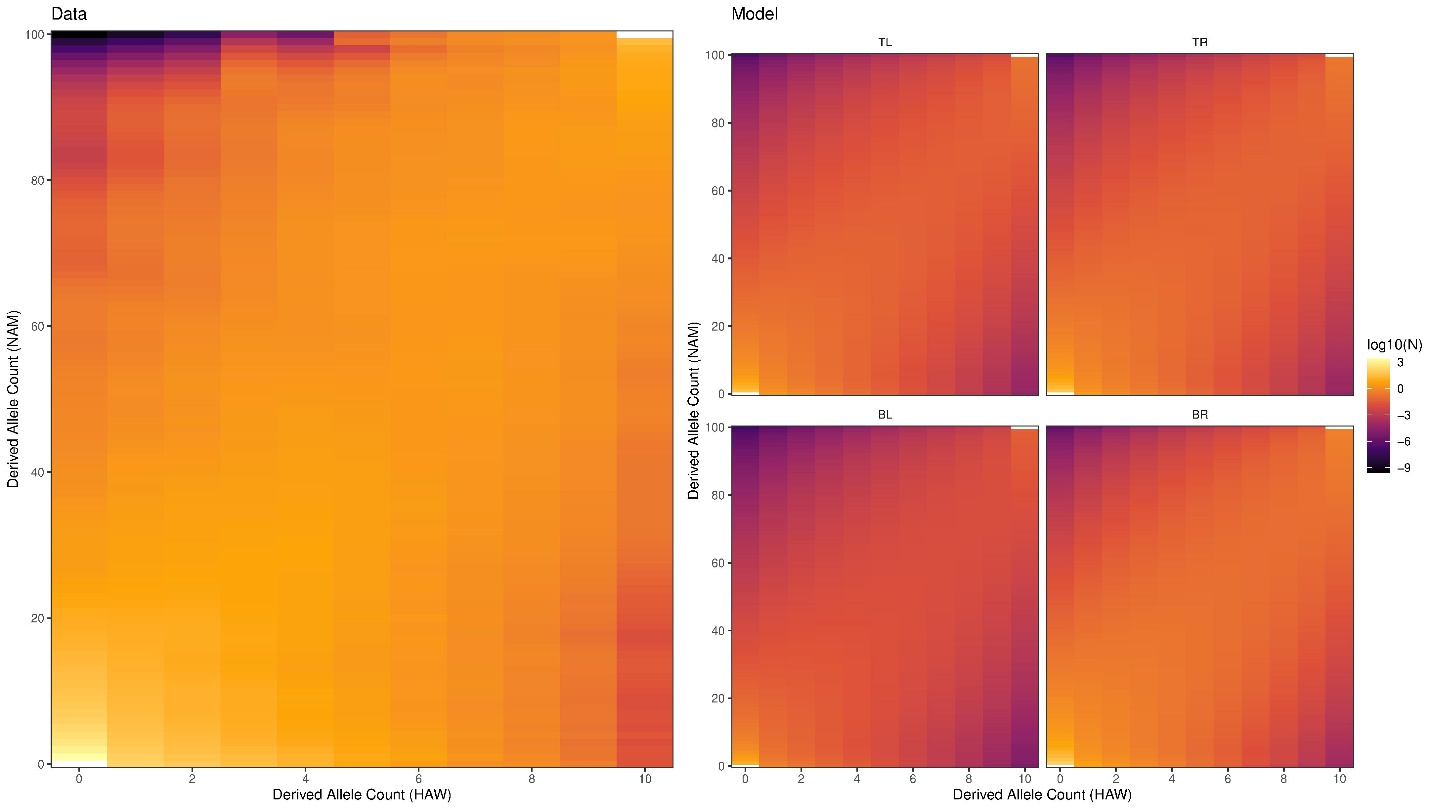


**Figure S7–** Observed (Left) and estimated (Right) derived site frequency spectra for the *Zhan* model. Cell brightness corresponds to the number of loci with derived allele frequencies in the given bin for both Hawaii (HAW) and North America (NAM). Estimated spectra based on the parameters from the runs with the lowest AIC score from each quadrant of the establishment time/founding population size parameter space for each model are shown for comparison (BL: bottom left, BR: bottom right, TL: top left, TR: top right), corresponding to the points marked in red in Figure 3.


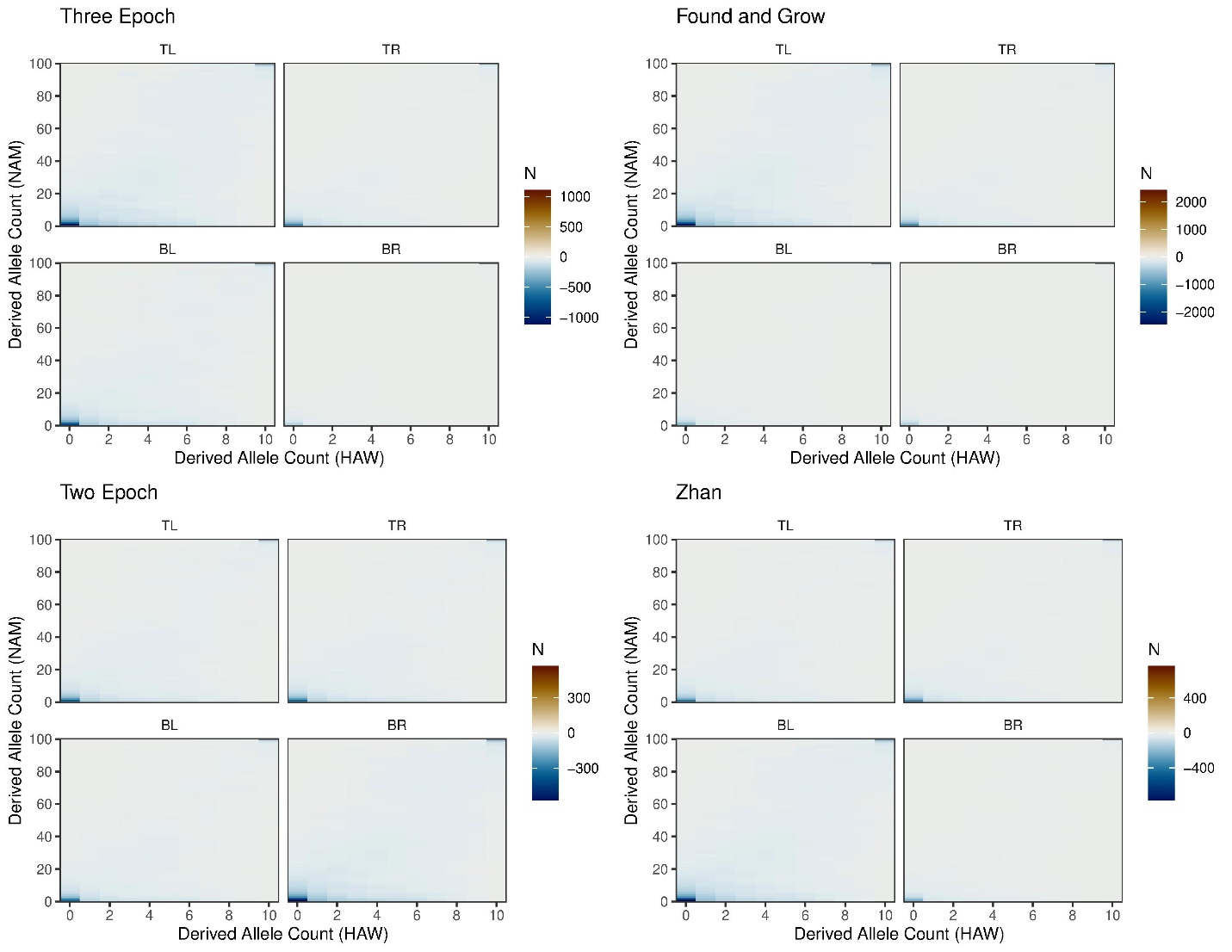


**Figure S8 –** Heatmaps of the residuals for optimized dadi parameter sets for the *Three Epoch , Found and Grow*, *Two Epoch*, and *Zhan* models. Cell colors correspond to the residual (model estimated - observed data) number of loci with derived allele frequencies in the given bin for both Hawaii (HAW) and North America (NAM). Optimized parameters from the runs with the lowest AIC score from each quadrant of the establishment time/founding population size parameter space for each model are shown for comparison (BL: bottom left, BR: bottom right, TL: top left, TR: top right), corresponding to the points marked in red in Figure 3.
